# Both Human Hematoma Punctured from Pelvic Fractures and Serum Increase Muscle Resident Stem Cells Response to BMP9: A Multivariate Statistical Approach

**DOI:** 10.3390/jcm9041175

**Published:** 2020-04-19

**Authors:** Yasaman Alinejad, Marc-Antoine Lauzon, Guillaume Grenier, Frédéric Balg, Nathalie Faucheux

**Affiliations:** 1 Laboratory of Endovascular Biomaterials (LBeV), Centre de recherche du CHUM (CRCHUM), 900 Saint-Denis Street, Montreal, QC H2X 0A9, Canada; Yasaman.Alinejad@USherbrooke.ca; 2 Department of Mechanical Engineering, École de Technologie Supérieure (ETS), 1100 Notre-Dame West, Montreal, QC H3C 1K3, Canada; 3 Advanced Dynamic Cell Culture Systems Laboratory, Department of Chemical and Biotechnology Engineering, Faculty of Engineering, Université de Sherbrooke, Sherbrooke, 2500 boul Université, Sherbrooke, QC J1K 2R1, Canada; Marc-Antoine.Lauzon@USherbrooke.ca; 4 Research Center on Aging, 1036, rue Belvédère Sud Sherbrooke, Sherbrooke, QC J1H 4C4, Canada; 5 Clinical Research Center of the Centre Hospitalier Universitaire de l’Université de Sherbrooke, 12e avenue Nord, Sherbrooke, QC J1H 5N4, Canada; Guillaume.Grenier@USherbrooke.ca (G.G.); Frederic.Balg@USherbrooke.ca (F.B.); 6 Department of Orthopedic Surgery, Faculty of Medicine, Université de Sherbrooke, 12e avenue Nord, Sherbrooke, QC J1H 5N4, Canada; 7 Laboratory of Cell-Biomaterial Biohybrid Systems, Department of Chemical and Biotechnological Engineering, Faculty of Engineering, Université de Sherbrooke, Sherbrooke, 2500 boul Université, QC J1K 2R1, Canada

**Keywords:** serum, cytokines, bone morphogenetic protein, alkaline phosphatase, human muscle resident mesenchymal stromal cells

## Abstract

Hematoma and skeletal muscles play a crucial role in bone fracture healing. The muscle resident mesenchymal stromal cells (mrSCs) can promote bone formation by differentiating into osteoblasts upon treatment by bone morphogenetic proteins (BMP), such as BMP9. However, the influence of hematoma fracture extracts (Hema) on human mrSC (hmrSC) response to BMP9 is still unknown. We therefore determined the influence of Hema, human healthy serum (HH), and fetal bovine serum (FBS, control) on BMP9-induced osteoblast commitment of hmrSC by measuring alkaline phosphatase activity. Multiplex assays of 90 cytokines were performed to characterize HH and Hema composition and allow their classification by a multivariate statistical approach depending on their expression levels. We confirmed that BMP9 had a greater effect on osteoblastic differentiation of hmrSCs than BMP2 in presence of FBS. The hmrSCs response to BMP9 was enhanced by both Hema and HH, even though several cytokines were upregulated (IL-6, IL-8, MCP-1, VEGF-A and osteopontin), downregulated (BMP9, PDGF) or similar (TNF-alpha) in Hema compared with HH. Thus, hematoma may potentiate BMP9-induced osteogenic differentiation of hmrSCs during bone fracture healing. The multivariate statistical analyses will help to identify the cytokines involved in such phenomenon leading to normal or pathological bone healing.

## 1. Introduction

When the bone is fractured, blood vessel rupture occurs leading to the formation of a hematoma. Such hematoma promotes the initiation of the inflammatory cascade due to the release of cytokines which favor chemotactic effect on inflammatory cells (neutrophils and macrophages) [1,2,3]. These cytokines are also involved in the recruitment of mesenchymal stem cells as well as progenitors of endothelial cells that colonize and proliferate into the hematoma. The mesenchymal stem cells can then differentiate into chondroblasts and osteoblasts under the action of the members of the TGF beta family especially the bone morphogenetic proteins (BMP) leading to endochondral bone fracture healing [4,5].

Skeletal muscles play also a crucial role in the bone fracture healing process. Several studies using animal models have shown that musculocutaneous coverage improves the open tibial fracture healing [6,7]. However, the underlying mechanisms by which skeletal muscles contribute to fracture healing are still poorly understood. The mesenchymal compartment of the skeletal muscle, especially the muscle resident mesenchymal stromal cells (mrSC), appeared to be involved in this phenomenon [8]. mrSCs have therefore received considerable attention as promising cells to develop strategies for improving bone fracture healing, mostly because of their capacity for BMP-dependent osteogenic differentiation [9,10,11,12]. For example, we recently found that in the presence of fetal bovine serum (FBS), BMP9 is a more potent osteoinductive factor on murine mrSCs than BMP2 [13]. However, the impact of fracture hematoma on human mrSC (hmrSC) fate toward osteoblastic lineage in response to BMP especially BMP9 has not been studied yet.

In the present study, we therefore investigated for the first time whether hematoma fracture extracts (Hema) and human healthy serum (HH) can promote the osteogenic differentiation of hmrSCs (CD31(−) CD34(−) CD90(−) CD73(+) CD105(+) CD140(−)) induced by BMP9. Such knowledge will help to develop new strategies to improve bone fracture healing using hematoma enriched by hmrSCs with BMP9. We have also used a principal component analysis (PCA) performed on 90 cytokines, after their quantification in HH and Hema samples by ELISA multiplex assays, to allow their classification into three clusters depending on significant differences between samples. Such experimental approach using PCA will be therefore helpful to identify the cytokines or growth factors that can be involved in hmrSCs fate not only in normal bone repair but also in pathological situations such as heterotopic ossification.

## 2. Experimental Section

### 2.1. Materials

PE-Cy7 anti-human CD31, clone WM59 (eBioscience) was purchased from Thermo Fisher Scientific (Ottawa, ON, Canada), while other antibodies (PE anti-human CD34, clone 581; FITC anti-human CD90; clone 5E10; APC anti-human CD73, clone AD2; PerCP-CyTM 5.5 anti-human CD105, clone 266; BV421 anti-human CD140a, clone αR1) and BD CompBeads Set Anti-Mouse Ig, κ were purchased from BD Biosciences (Mississauga, ON, Canada); BMP2 and BMP9 were purchased from R&D Systems (Minneapolis, MN, USA); Collagenase type I, ascorbic acid, dexamethasone and β-glycerophosphate were purchased from Sigma-Aldrich, (Oakville, ON, Canada); Fetal bovine serum (FBS, Hyclone), Horse serum, Glutamax (Gibco), DMEM, LIVE/DEAD^®^ Violet Viability Kit (Invitrogen), Penicillin/Streptomycin (*p*/S) were purchased from Thermo Fisher Scientific; Mesencult-XF^®^ medium, Mesencult-XF^®^ human supplement and Mesencult-XF^®^ attachment substrate were purchased from StemCell Technologies (Vancouver, BC, Canada); SensoLyte^®^ pNPP Alkaline Phosphatase Assay Kit was purchased from AnaSpec (Fremont, CA, USA).

### 2.2. Patient Recruitment Protocol and Sample Conditioning

The orthopedic and traumatology department at the Hospital Center of University of Sherbrooke (CHUS) recruited male and female patients from 18 to 80 years of age admitted for traumatic pelvic fractures requiring surgery (Table 1). Patients who received plasma or blood transfusion upon admission to the hospital or those with pathologic fractures (e.g., cancer or known osteoporosis condition) or pregnant were rejected. Following the ethic protocol conducted according to the Declaration of Helsinki and the rules of ethics committee of CHUS (#11-063), the purpose of the research and the protocol was explained to the consenting patients whose condition corresponded to the inclusion criterion. For each patient recruited, blood samples (2 × 8 mL tubes) were taken during the surgery (hematoma punctured). Upon reception, hematoma samples were centrifuged in order to keep the serum portion only and were frozen at −80 °C. Blood sample from healthy patients were taken from consenting researchers and research assistants working at the Center for Clinical Research at the CHUS (22–43 years of age without chronic disease or plasma/blood transfusion, no pregnant woman) (Table 1). Blood samples were pretreated using the same protocol (i.e., resting for 30 min at RT followed by centrifugation at 1200 g for 15 min, collecting the upper part and immediately freezing at −80 °C).

### 2.3. Human Muscle Resident Stromal Cells Harvesting, Sorting and Cultivation Protocol

Human muscle resident stromal cells (hmrSCs) were extracted from fresh skeletal muscle samples (gracilis and semitendinosus) obtained from patients (mean age, 32 years; range, 18 to 48 years; 54% male and 46% female) undergoing anterior cruciate ligament reconstruction surgery (Ethics protocol #11-122). Detailed information of the patients can be found in our previous publication [10]. Briefly, the muscle piece was digested in 1 mg/mL collagenase type I for 30 min at 37 °C, passed through cell strainer and centrifuged (325× *g*, 6 min, 4 °C). Cell pellets were resuspended in Mesencult-XF^®^ medium containing 20% (v/v) Mesencult-XF^®^ human supplement, 1% (v/v) Glutamax and 1% (v/v) *p*/S, a mixture called defined medium throughout the study. The cells were seeded in a 100 mm culture plate coated with Mesencult-XF^®^ attachment substrate and incubated at 37 °C. At 80% confluence, cells were trypsinized, centrifuged (325× *g*, 6 min, 4 °C), and resuspended for FACS separation at ~1 × 10^6^ cells/mL in the presence of appropriate fluorescent labeled primary antibodies against CD31, CD34 CD90, CD73, CD105, and CD140. The LIVE/DEAD^®^ Violet Viability Kit was used to separate live cells from dead ones, whereas fluorescence compensation was evaluated using the BD CompBeads Set Anti-Mouse Ig, κ as previously published [10]. A BD FACS Aria™ cell sorter (BD Biosciences) equipped with four lasers and a 100-μm nozzle was used at 20 psi and sorted cells were analyzed by FlowJo 7.9 software (Treestar Inc., Ashland, OR, USA). Sorted cells (CD31(−) CD34(−) CD90(−) CD73(+) CD105(+) CD140(−) hmrMSCs) were expanded in the defined medium on coated tissue culture dishes for future cell culture experiments.

### 2.4. Alkaline Phosphatase Activity Assays

Following incubation, hmrSCs were washed twice with sterile phosphate buffered saline (PBS), and then incubated for 20 min with Hoechst dye (5 µg/mL in sterile PBS). At least 4 pictures at low magnification (0.03 cm^2^/pictures) per experimental conditions and per replicate were randomly taken using a Leica epifluorescence microscope (DMIRE2 inverted microscope, Leica Microsystems). Cell density was determined using a cell counting application programmed in Matlab (2014b, MathWorks) as previously reported [13,14]. ALP activity was measured after cell lysis using SensoLyte^®^ pNPP Alkaline Phosphatase Assay Kit following the manufacturer’s instructions. The concentration of ALP was determined with a standard curve using the ALP standard solution provided with the assay kit. The concentration of ALP was normalized in respect to the average cell density and then compared with the control.

### 2.5. Dose Response of BMP2 and BMP9 and EC_50_ Determination

hmrSCs were cultured on collagen-coated (0.1 mg/mL, Millipore) tissue culture-treated 6-well plates until they reached 80% of confluence. Cells were then stimulated for 3 days (time required to observe ALP activity as an indicator of osteoblastic differentiation [13]) with various concentrations of BMP2 (0, 0.1, 0.2, 0.4, 1, 1.92, 4, 8, 12 and 20 nM) and BMP9 (0, 0.01, 0.05, 0.1, 0.2, 0.4, 1.92, 4 and 8 nM) in cell culture media containing 10% (*v/v*) FBS. From experimental data, EC_50_ was estimated using the following mathematical expression where RALP represent the relative ALP, [BMP] stands for the concentration of BMP and beta (β) represents the Hill coefficient, which give the largest absolute value of the slope of the curve:

(1) $$RALP=RALP_{min}+\frac{\left(RALP_{max}-RALP_{min}\right)}{1+{\left(\frac{\left[BMP\right]}{EC_{50}}\right)}^{-\beta }}$$

The value of RALP_max_, β and EC_50_ were estimated from the experimental data using a meta-heuristic algorithm (genetic algorithm) [13,15]. Metaheuristic algorithms are powerful optimization tools when parameters to estimate have sensitivity discrepancies, when the objective function has many local optima or when it is difficult to differentiate [16]. The confidence intervals (CI) of each model parameter were estimated using a bootstrapping methodology. Briefly, residual values between experimental and best-fit data were randomly picked to add to the model data in order to generate 500 new data sets. Using the same optimization algorithm, distribution of the model parameters was estimated. The 95% confidence intervals were defined as

(2) $$CI=\pm 1.96\frac{\sigma }{\sqrt{n}}$$

### 2.6. Dose Response of Serum from Healthy Human on hmrSCs with or without BMP9

hmrSCs were cultured on collagen-coated (0.1 mg/mL, Millipore) tissue culture-treated 6-well plates until they reached 80% of confluence. Cells were then stimulated for 3 days with different concentrations of serum from healthy human (0, 1, 5 and 10% *v*/*v*) in culture media containing 1.92 nM of BMP9. To confirm HH effect, hmrSCs were also incubated with HH from 6 patients (5% *v*/*v*) with BMP9 at 0.1 nM, a concentration below its EC_50_ to detect any additive or synergistic effect with HH. Osteogenic media (OS), containing DMEM supplemented with 5% (*v*/*v*) horse serum, 1% (*v*/*v*) *p*/S, 10 µg/mL ascorbic acid, 100 nM dexamethasone and 10 mM β-glycerophosphate, was used as a positive control. Horse serum was used instead of FBS because of its lower growth factor content, when the cell growth was not required but osteogenic differentiation was evaluated in the presence of specific osteoinductive factors. Relative ALP activity was assessed as previously described.

### 2.7. Multiplex ELISA Assays

Serum samples were diluted using standard PBS pH~7.5 prior to shipping for a multiplex analysis. Serum samples were tested by Eve Technologies (Calgary, AB, Canada) for several cytokine assays: (i) Human Angiogenesis 17-plex, (ii) Human bone 6-plex, human cytokine 64-plex, (iii) Human supplemental 10-plex, and (iv) Human TGFβ 3-plex. Overall, 90 different cytokines were assessed.

### 2.8. Correlation Matrix, Statistical Analysis, Principal Component Analysis and Clustering of Cytokines Multiplex ELISA Assay Results

Cytokines quantification data from multiplex ELISA assays were mean centered. Correlation matrix between each patient sample was determined using the following equation in order to validate whether there were correlations between and within patient groups.

(3) $$M_{corr}=\frac{1}{n}X_{s}^{T}X_{s}$$

where

$X_{s}=CXD^{-1}$ = covariance matrix
X = non-processed raw data
$C=I_{n}-n^{-1}1_{n}1_{n}^{T}$ with I*_n_* the identity matrix and 1*_n_*, a column vector of “*n*” ones
$D=diag\left(S_{1}\ldots \;S_{n}\right)$ with *S* the standard deviation

Cytokines quantification data from multiplex ELISA assays were also assessed by a principal component analysis (PCA). The PCA is a dimensionality reduction technic consisting in a statistical procedure that uses orthogonal transformation of the data converting variables to linearly uncorrelated ones called principal components (PC). Each principal component is composed of a vector of observations (Scores), corresponding to the projections of original data on the new axe and a vector of weights (Loadings), representing the uncorrelated variables in this new axe. This technique can help to identify and classify groups of observations in reduced dimension space. Because the concentration results of the tested cytokines had an overall range of several orders of magnitude, original data were reduced-centered in order to assign a similar relative scale to each parameter. Scores and loadings were determined using the *Nonlinear Iterative partial Least Squares* (NIPALS) algorithm combined with a Gram-Schmidt reorthogonalization step. Data were also checked for outliers using Hotelling’s T-squared and Q_residuals_ tests.

Following the PCA analysis, *Expectation-Maximization Clustering Algorithm* (EMCA) was also applied to cluster the loadings into distinctive groups based on their probability to be part of a given group. EMCA aims to assign *n* observations (scores) into “*k”* cluster distributions to which it has the maximum probability to be part of (with a number of groups known a priori by the user). The cluster distribution is corrected by the Bayes theorem.

Next, a statistical analysis was performed to determine the cytokines, belonging to a specific cluster, that were significantly different between patient groups using paired Student’s *t*-test. In these tests, each patient from a defined group was considered as a replicate.

Finally, statistical analysis other than those related to multiplex assays were assessed on Microsoft Excel (Analysis tool pack) by means of analysis of variance (one way or two-way ANOVA) followed by Tuckey post-hoc pairwise comparison test. Only differences with *p* < 0.05 were considered statistically significant.

### 2.9. Effect of Osteopontin on hmrSCs Cells Response to BMP9

hmrSCs were cultured on collagen-coated (0.1 mg/mL, Millipore) or collagen [0.1 mg/mL] + OPN [5 µg/mL])) tissue culture-treated 6-well plates until they reached 80% of confluence. Cells were then stimulated for 3 days with culture media containing 0.1 nM BMP-9 with (5% *v*/*v*) or without serum from HH. Relative ALP activity was assessed as previously described.

## 3. Results

### 3.1. Effect of BMP on hmrSCs Differentiation with or without Serum or Hematoma

#### 3.1.1. Dose Response of BMP2 and BMP9 and EC_50_ Determination in the Presence of Fetal Bovine Serum

**Figure 1:**
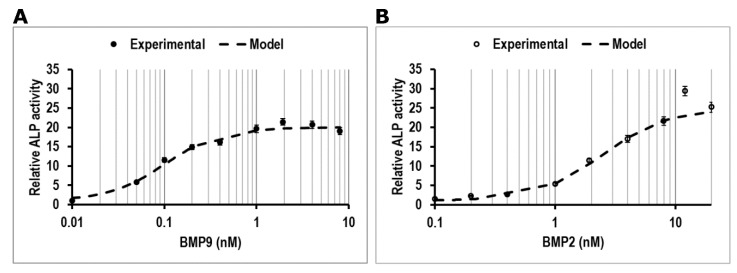
ALP activity measurement relative to control in respect to the number of cells. Dose-response effect of (**A**) BMP9 (**●**), (**B**) BMP2 (**○**) on ALP activity in hmrSCs incubated for 3 days in culture medium containing 10% (*v*/*v*) FBS and their mathematical modeling (**-**). The results are the mean ± SEM of four independent experiments performed in triplicate.

To confirm whether hmrSCs (CD31(−) CD34(−) CD90(−) CD73(+) CD105(+) CD140(−)) react similarly to murine mrSCs to BMP2 and BMP9 in the presence of FBS in terms of relative ALP activity [13], cells were stimulated for 3 days with BMP2 (0, 0.1, 0.2, 0.4, 1, 1.92, 4, 8, 12 and 20 nM) or BMP9 (0, 0.01, 0.05. 0.1, 0.2, 0.4, 1, 1.92, 4 and 8 nM) in the presence of 10% (*v*/*v*) FBS. Dose-response data (Figure 1A,B) showed a typical sigmoidal shaped curve for both BMP2 and BMP9. The cells were more sensitive to BMP9 than BMP2 as a lower concentration was required to increase the relative concentration of ALP as confirmed by mathematical modeling of the dose-response (dashed lines on Figure 1A,B) (Table 2). The EC_50_ for BMP9 was ~42-fold lower compared to BMP2 (0.09 nM BMP9 vs. 3.75 nM BMP2). Parameters’ estimation using the metaheuristic genetic algorithm also showed that the Hill coefficient (β) values were similar for both BMPs, thus indicating that the maximum slope observed were mostly equivalent. However, 12 times more BMP2 was required to reach the plateau (12 nM) compared with BMP9 (1 nM).

#### 3.1.2. Dose response of Serum from Healthy Human on hmrSCs with or without BMP9

**Figure 2:**
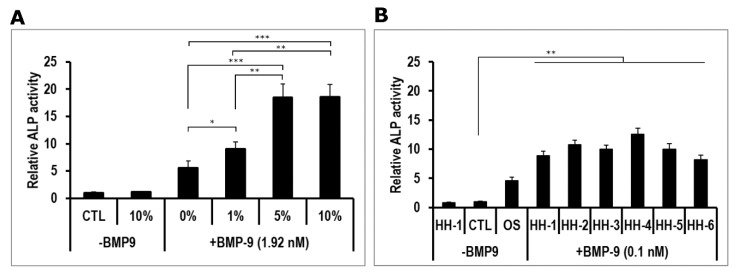
(**A**) Dose-response effect of healthy human serum (HH-1) on ALP activity in hmrSCs incubated for 3 days with or without 1.92 nM BMP9. Results are mean ± SEM of 4 independent experiments performed in triplicate. (**B**) Comparison of several HH (5% *v*/*v*) on ALP activity in hmrSCs incubated for 3 days with or without 0.1 nM BMP9. Results are mean ± SEM of 4 independent experiments performed in triplicate. * *p* < 0.05, ** *p* < 0.01, *** *p* < 0.001.

Then, to confirm whether human serum acted on hmrSCs response to BMP9 as did FBS, cells were stimulated with various concentrations of serum from a healthy donor (HH-1) with or without BMP9 at 1.92 nM. BMP9 was used at this concentration since it induced the maximum value of relative ALP activity in the presence of FBS (positive control) at 10% (*v*/*v*) (Figure 2A). Without BMP9, the relative ALP activity was similar in the presence (10% *v*/*v*) or in the absence (CTL) of HH-1 serum. In the presence of serum, a dose response was observed from 0% to 5% (*v*/*v*), where it reached a plateau. Since there were no significant difference between 5% and 10% (*v*/*v*), a concentration of 5% (*v*/*v*) of human serum was kept for the rest of the study. The maximum value of relative ALP activity measured was quite similar to what was observed with FBS.

The effect of HH on hmrSCs response to BMP9 was further verified by using additional serum samples from five healthy donors (HH-2 to HH-6). The cells were stimulated for 3 days with HH with or without BMP9 used at its EC_50_ (0.1 nM) to verify whether HH can enhance the BMP9 cell response (Figure 2B). As a positive control, cells were also stimulated with osteogenic medium (OS). Without BMP9, the relative ALP activity was similar in the presence (5% *v*/*v*) or in the absence (CTL) of HH-1 serum. Osteogenic medium significantly increased (*p* < 0.001) the level of relative ALP activity compared to the control and serum from HH-1 alone. With BMP9, the values of relative ALP activity in the presence of HH serum (HH-1 to HH-6) were all significantly higher than all other conditions (*p* < 0.01). There were no significant difference nor important variability between the HH serums.

#### 3.1.3. Effect of Human Hematoma on hmrSCs Response to BMP-9

**Figure 3:**
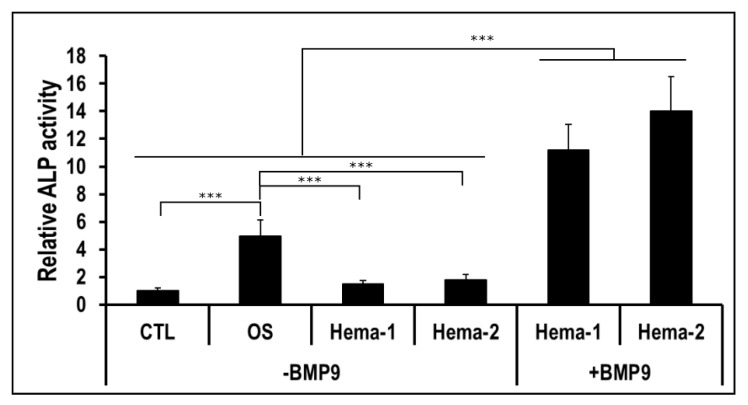
ALP activity relative to the control in respect to the cell number. hmrSCs were stimulated for 3 days with serum from hematoma of 2 patients (Hema-1 and Hema-2) with or without 0.1 nM BMP9. Osteogenic culture medium (OS) was used as a positive control. The results are the mean ± SEM of two independent experiments performed in triplicate. ***p < 0.001.

We then determined whether the Hema punctured from pelvic fracture could also potentiate the hmrSCs cell response to BMP9 as did HH sera. hmrSCs were stimulated for 3 days with punctured hematoma (Hema) samples of two patients with or without BMP9 (0.1 nM) (Figure 3). The Hema samples alone did not show any significant impact on cell expression of ALP as compared with the CTL. However, the presence of BMP9 drastically increased (*p* < 0.001) the relative ALP expression by 7.5-fold. The combination between BMP9 and Hema was also significantly higher (2.5-fold) than the osteogenic culture media (OS) (*p* < 0.001). These results indicate that the hematoma can also potentiate drastically the hmrSCs response to BMP9.

### 3.2. Multiplex Analyses of Cytokines in Healthy Human Serum and Hematoma

#### 3.2.1. Correlation Analysis

**Figure 4:**
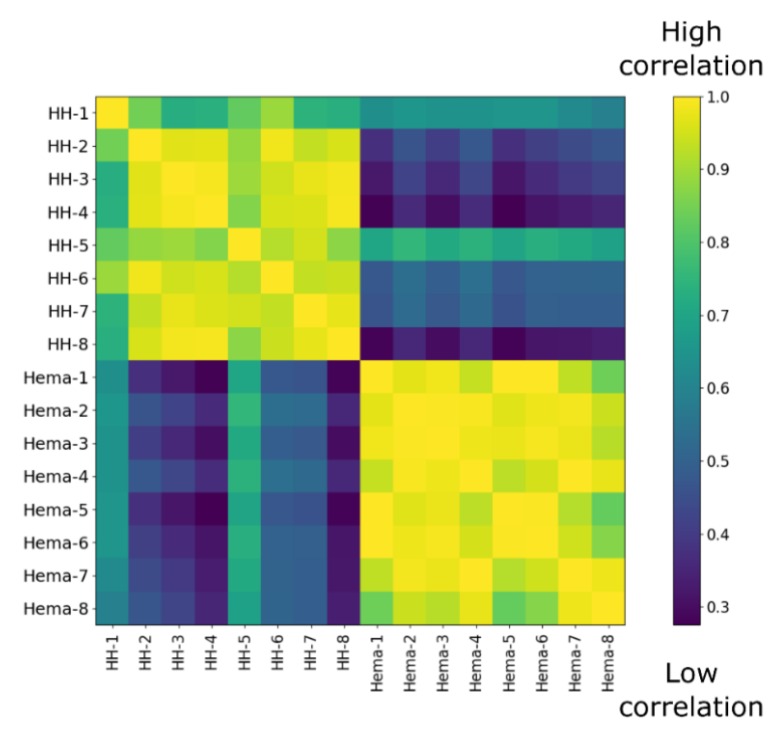
Correlation matrix of ELISA multiplex data from 8 healthy donor serum (HH-#) versus 8 hematoma extracts from pelvic fractures (Hema-#).

Since we did not observe any difference between sera from healthy patient and Hema extracts in terms of osteogenic induction, we next studied more thoroughly the cytokine compositions as the hematoma is usually rich in inflammatory, osteogenic and angiogenic factors [3,17,18]. The concentrations of over 90 cytokines from the sera of 8 healthy humans (HH-#) and the punctured hematoma from eight pelvic fractures (Hema-#) were assessed by multiplex ELISA assays. From those results, we first performed a correlation analysis. From the resulting heat map (Figure 4), a clear difference between HH serum and Hema could be observed as the correlation among the groups were very high (>0.75), but very low between groups (<0.6). We also compared the results in respect to the sex and age of the patients (supplementary data, Figure S1). However, we did not observe any tendency.

#### 3.2.2. Principal Component Analysis and Clustering

**Figure 5:**
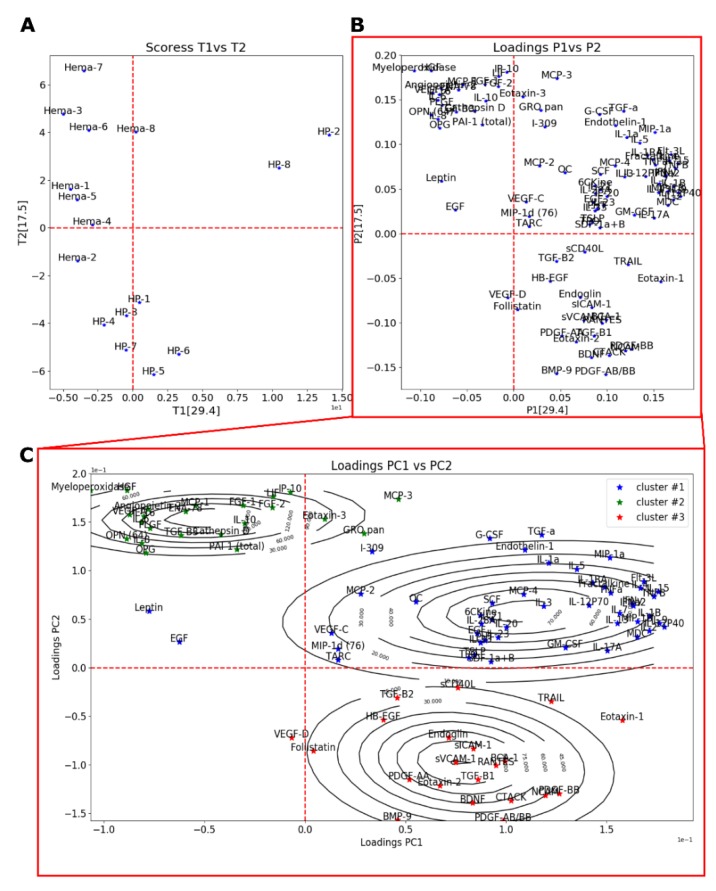
PCA analysis of ELISA multiplex cytokine dosage showing the scores depicted as individuals (**A**) and the loadings depicted as the cytokines (**B**) of PC1 (29.4% variance explained) and PC2 (17.5% variance explained). (**C**) Results of clustering of loadings on PC1 and PC2 using Expectation-Maximization Clustering Algorithm showing the loadings (stars) and the 2D estimated distribution (contour lines).

In order to investigate this inter-group difference and since many cytokines were studied simultaneously, a PCA was performed on the centered-reduced data (Figure 5A,B). The first principal component (PC1) could explain ~29.4% of the variance, whereas PC2 explained ~17.5%. The variation on PC1 appeared to be caused by serum sample of two healthy patients (HH-2 and HH-8), while the variation on PC2 was mostly explained by the difference between hematoma (Hema-#) and HH (HH-#) samples. Looking at the loading plots (PC1 vs. PC2), distinctive groups having a great impact on the PC2 could be observed. To affine our research, we performed a clustering analysis using the *Expectation-Maximization Clustering Algorithm*. In this study, each parameter was assigned statistically to a distinctive group (Figure 5C). *Cluster #2* and *cluster #3* regroup cytokines that contribute the most to the variance observed between serum from healthy patients (HH-#) versus hematoma (Hema-#). Cytokines belonging to *cluster #2* had a positive effect on Hema samples, meaning that there was an increase in their values, whereas cytokines belonging to *cluster #3* had a decrease in their values. Remaining cytokines belonging to cluster #1 only had a small contribution to the difference observed between our two patient groups (Hema and HH).

Based on the clustering algorithm results, the contribution of the cytokines belonging to each cluster was more thoroughly studied (paired Student’s comparison test) in order to determine which cytokines had the most significant difference between HH and Hema samples. Cytokines belonging to the *cluster #1* were all non-significant (Table S1). Those results were expected from the PCA analysis, as their position on the PC1 vs. PC2 loading plot (Figure 5C) indicated that they had a similar contribution in HH and Hema samples. Table 3 shows the statistical significance as well as the observed effect relative to the Hema results of cytokines belonging to *cluster #2* and *cluster #3*. From the pair wise statistical analysis of *cluster #2* and *cluster #3*, the difference in the protein levels of several cytokine families was found to be highly significant. Many cytokines related to inflammation and activated immune system were more expressed in Hema sample (from *Cluster #2*: MCP-1, *p* < 0.05; Myeloperoxidase, *p* < 0.001; IL-6, *p* < 0.05; IL-8, *p* < 0.05; IL-10, *p* < 0.05; IL-16, *p* < 0.01) as well as many pro-angiogenic and pro-endothelial agents (from *Cluster #2*: Angiopoietin-2, *p* < 0.01; HGF, *p* < 0.001; FGF-2, *p* < 0.05; VEGF-A, *p* < 0.05), or platelet associated growth factors (PLGF, *p* < 0.01). Aside from those cytokines, the osteogenic factor OPN was also found to be highly present in Hema sample (*p* < 0.001). Those results are in accordance with the PCA observations (PC2). Interestingly, BMP9 (*p* < 0.01), BDNF (*p* < 0.01) and PDGF related growth factors (PDGF-AA *p* < 0.01; PDGF-AB/BB, *p* < 0.01; and PDGF-BB. *p* < 0.001) were significantly lower (*Cluster #3*). In fact, there were mostly no BMP9 measured in Hema sample (0.43 ± 0.43 (SEM) pg/mL), whereas an average concentration of 27.42 ± 6.33 pg/mL was found in HH serum.

#### 3.2.3. Effect of Osteopontin on Human Muscle Resident Stem Cells Response to BMP-9 in the Presence of HH

**Figure 6:**
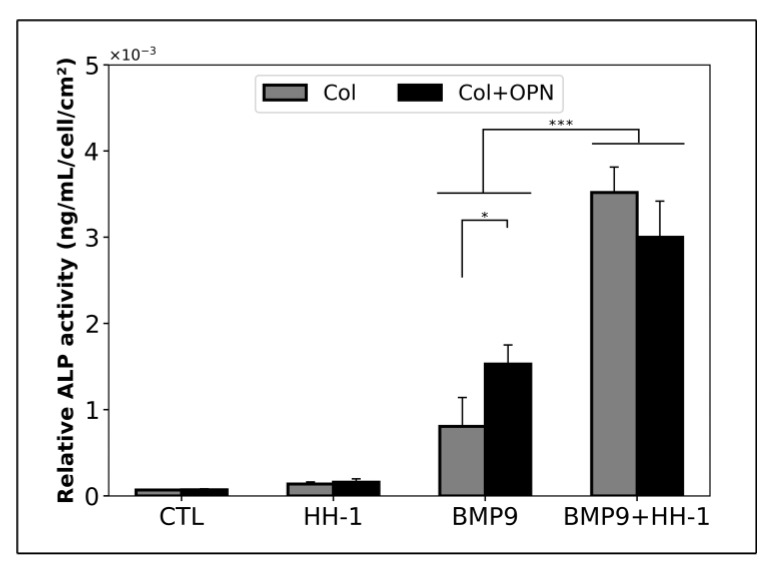
ALP activity relative to the cell number. hmrSCs were cultured on a collagen coating (Col) or a collagen and osteopontin (Col (0.1 mg/mL) + OPN (5 µg/mL)) coating and stimulated for 3 days with 0.1 nM BMP9 with or without HH serum (HH-1). The results are the mean ± SEM of 2 independent experiments performed in triplicate. * *p* < 0.05, *** *p* < 0.001.

As OPN increased significantly in the Hema sample, while BMP9 significantly decreased, we next determined the impact of OPN coating on the capacity of hmrSCS to express ALP with or without BMP9. For this purpose, ALP activity was measured in hmrSCs cultured on polystyrene coated with collagen (Col) or with Col (0.1 mg/mL) + OPN (5 µg/mL) and stimulated for 3 days with or without BMP9 (0.1 nM) in the absence or presence of HH-1 (5% *v*/*v*) (Figure 6). Without BMP9, the ALP activity referred to the cell density was similar in hmrSCs adherent to Col or Col + OPN in serum free medium or HH-1. With BMP9, the ALP activity was higher in serum free medium in hmrSCs adherent to Col + OPN in comparison to Col (*p* < 0.05). However, Col + OPN did not increase the hmrSC response to BMP9 compared with Col alone when the cells were cultured with HH-1.

## 4. Discussion

Both hematoma and skeletal muscles were found to play a crucial role in bone fracture healing [19,20]. For example, using a periosteally stripped open tibial fracture model in mice, Glass et al. found that cells isolated from muscles adjacent to the fracture site are positively stained for ALP [8]. We have previously found that hmrSCs were potent stem cells capable of differentiation toward chondrogenic and osteogenic lineages [10]. hmrSCs were also highly responsive to BMP9 in vitro [10]. In this study, we investigated for the first time the effect of HH and Hema on hmrSCs cell response to BMP9 that will help to develop new strategies to improve bone fracture healing or better understand mechanisms underlying pathologic state in injured musculoskeletal tissue. We showed that hmrSCs were more responsive to BMP9 than BMP2 in the presence of FBS. The dose-response experiments and mathematical modeling showed that the EC_50_ for BMP9 was significantly lower (~4-fold) than that of BMP2. Unlike BMP2, EC_50_ and maximum concentration values obtained for BMP9 were in the range of BMP physiological concentration (0 to 4 nM) found in serum [21]. Those results are in accordance with our previous studies performed on murine preosteoblasts and murine mrSCs from normal and damaged muscles, where we demonstrated that FBS increased significantly the cell response to BMP9 in opposition to BMP2 [13,14]. Based on those findings, we verified whether hematoma punctured from pelvic fracture (Hema) could increase the hmrSC response to BMP9. We showed that Hema combined with BMP9 could increase significantly the ALP activity in hmrSCs compared with the control or the osteogenic medium. The effect of Hema on hmrSCs response to BMP9 was similar to that of human healthy serum (HH).

In order to determine whether the composition of Hema was different from HH, we then assessed the concentrations of 90 cytokines by multiplex assay in order to identify some biomolecules that may explain the response of hmrSCs in the presence of BMP9 plus Hema. Combining advanced dimension reduction analysis tools such as correlation analysis, PCA and clustering algorithm proved to be very efficient to discriminate cytokines of interest. Our results highlighted the relevance of using such tools designed to extract meaningful elements. This approach should be used more often to analyze such a data set. For instance, the correlation analysis showed that all HH and Hema samples were highly correlated together in their distinctive groups, but only shared low correlation one group toward another. The principal component analysis indicated that many cytokines related to inflammation and activated immune system (myeloperoxidase, Il-6, Il-8 and MCP-1) as well as many angiogenic factors (angiopoetin-2, VEGF-A) were found to be significantly more expressed in hematoma. Those observations are in accordance with the literature, where fracture hematoma composition is known to have high levels of IL-6, IL-1, MCP and proangiogenic factors [3,17,18,22]. For instance, Hoff et al. observed increased level of IL-6, IL-8, MCP-1 and VEGF in early punctured fracture hematoma (<72 h) from patients undergoing total hip arthroplasty surgery [17]. Pountos et al. recently found that 33 inflammatory cytokines like IL-8 and IL-11 as well as metalloproteinases MMP1, MMP2 and MMP3 were increased in fracture hematoma in comparison to peripheral serum. The level of anti-inflammatory cytokine IL-10 was also significantly enhanced in fracture hematoma compared to peripheral serum [19].

Interestingly, we found that the levels of the potent osteoinductive factor BMP9 were significantly lower in Hema of every patient (~0.4 pg/mL), in comparison to human serum (HH) (~27 pg/mL). We have already suggested that BMP9 may induce heterotopic ossification (HO). Given a permissive microenvironment such as an injury, mrSCs were highly sensitive to BMP9, and could lead to ectopic bone formation in mice [23]. Moreover, we reported a case where BMP9 was expressed in ectopic bone tissue from a patient who suffered extensive trauma [24]. However, Crowgey et al. recently found a decrease in BMP9 levels in serum samples from patients experiencing HO (7.9 pg/mL for patient whom had HO versus 10.4 pg/mL for patients with a non-pathological condition) [25]. Therefore, it might be interesting in a future study to compare the BMP9 levels in hematoma leading to normal and pathologic fracture healing or HO.

Surprisingly, despite strong differences in cytokine profiles in HH and Hema, both of them significantly promote the hmrSCs cell response to BMP9 in terms of osteogenic differentiation. For example, we found that some pro-osteogenic factors such as OPN were highly expressed in the Hema as compared to HH. Pountos et al. also recently found that OPN levels increased around 2.4-fold in fracture hematoma in comparison to peripheral serum [19]. OPN is a cytokine-like, calcium binding, and multifunctional protein playing a major role in the commitment of stem cells into osteogenic lineage in the presence of BMPs [26]. We therefore evaluated whether adding OPN coating could have any impact on hmrSC response to BMP9 in the presence of HH. OPN coating potentiated significantly the hmrSC cell response to BMP9 as measured by ALP activity. However, OPN did not have any significant impact on cell stimulated with HH with or without BMP9. Our results thus suggested that combining BMP9 with OPN in fracture hematoma may potentiate the osteogenic differentiation of hmrSCs.

Interestingly, both Hema and HH had also similar levels of TNF-alpha. Glass et al. found that TNF-alpha in the supernatants from human fractured tibial bone fragments promotes the osteogenic differentiation of muscle-derived stromal cells expressing CD73, CD90, CD105, and HLA-ABC in vitro [8]. Further experiments are therefore required to determine why both HH and Hema extracts promoted hmrSC response to BMP9 despite significant differences in their cytokine profiles.

## 5. Conclusions

In conclusion, we showed, in this study, that hematoma punctured from pelvic fracture can potentiate the hmrSCs response to BMP9. In addition, analysis of 90 cytokines content revealed a strong difference in the composition of the hematoma and healthy human serum. Some of the constituent, which are present in significantly higher concentrations in hematoma, such as osteogenic factor OPN, can potentiate the stem cell response to BMP9. While we have not found any BMP9 in hematoma of those fractures, our previous studies indicated that the presence of BMP9 in injured musculoskeletal tissue could lead to pathologic state. Thus, the next step would be to investigate how the composition of hematoma of patients who have developed pathological bone fracture healing or HO is affected, and if a similar approach using a multivariate statistical analysis could deepen our understanding of the role played by BMP9. Such knowledge could be also essential to develop a new strategy combining hmrSCs and BMP9 to favor bone repair.

## Figures and Tables

**Table 1:** Demographic information of patients.

Patients		Sex	Age	Patients		Sex	Age
**Serum from healthy human**	1	M	43	**Hematoma (pelvic fracture)**	1	F	33
	2	M	33		2	M	71
	3	F	36		3	M	79
	4	F	29		4	F	69
	5	F	35		5	F	21
	6	M	23		6	M	51
	7	M	33		7	F	23
	8	M	22		8	M	59
	**Average**	0.62(M)/0.38(F)	31.75		**Average**	0.5(M)/0.5(F)	50.75
	**SEM**	-	2.44		**SEM**	-	8.13

**Table 2:** Estimated mathematical parameters and modeling statistics ± confidence intervals.

Parameters	BMP2	BMP9
RALP_max_	31.25 ± 0.36	19.34 ± 0.32
Beta (β)	1.23 ± 0.05	1.97 ± 0.07
EC_50_	3.75 ± 0.09	0.09 ± 0.02
R^2^	0.98	0.98
Model statistics	*p* < 0.001	*p* < 0.001

**Table 3:** Statistical significance and effect of cytokines from multiplex ELISA assay (*cluster #2* and *cluster #3*) contributing the most to the variance observed between HH-# versus Hema-# patients.

Cytokines from Cluster #2	Significance	Effect	Cytokines from Cluster #3	Significance	Effect
Angiopoietin-2	*	↑	BCA-1	N. S	-
Cathepsin D	N.S.	-	BDNF	**	↓
ENA-78	N.S.	-	BMP-9	**	↓
Eotaxin-3	N.S.	-	CTACK	**	↓
FGF-1	N.S.	-	Endoglin	N.S.	-
FGF-2	*	↑	Eotaxin-1	*	↓
GRO pan	N.S.	-	Eotaxin-2	N.S.	-
HGF	***	↑	Follistatin	N.S.	-
IL-10	*	↑	HB-EGF	N.S.	-
IL-16	**	↑	NCAM	**	↓
IL-6	*	↑	PDGF-AA	**	↓
IL-8	*	↑	PDGF-AB/BB	**	↓
IP-10	N.S.	-	PDGF-BB	***	↓
LIF	N.S.	-	RANTES	N.S.	-
MCP-1	*	↑	sCD40L	N.S.	-
MCP-3	N.S.	-	sICAM-1	N.S.	-
Myeloperoxidase	***	↑	sVCAM-1	N.S.	-
OPG	N.S.	-	TGF-B1	N.S.	-
OPN	***	↑	TGF-B2	N.S.	-
PAI-1 (total)	N.S.	-	TRAIL	*	↓
PLGF	**	↑	VEGF-D	N.S.	-
TGF-B3	*	↑			
VEGF-A	*	↑			

Not significant (N.S.), Significant increase (↑) or decrease (↓) compared to HH serum (* *p* < 0.05, ** *p* < 0.01, *** *p* < 0.001).

## Supplementary Materials

The following are available online at https://www.mdpi.com/2077-0383/9/4/1175/s1, Table S1. Statistical significance and effect of cytokines from cluster #1. Figure S1. (A) Correlation matrix of ELISA multiplex data from serum from 8 healthy donors (HH-#) versus 8 hematoma extracts from pelvic fractures (Hema-#) organized in respect to their sex. (B) PCA analysis of ELISA multiplex cytokine dosage showing the scores depicted as individuals according to their sex. (C) Correlation matrix of ELISA multiplex data from 8 healthy donor serum (HH-#) versus 8 hematoma extracts from pelvic fracture (Hema-#) organized in respect to their age. (D) PCA analysis of ELISA multiplex cytokine dosage showing the scores depicted as individuals according to their age.
