# Supplementary material for: Both Human Hematoma Punctured from Pelvic Fractures and Serum Increase Muscle Resident Stem Cells Response to BMP9: A Multivariate Statistical Approach
Source: J Clin Med. 2020 Apr 19;9(4):1175. doi: 10.3390/jcm9041175 (PMC7231246; doi:10.3390/jcm9041175)
Supplement: Supplementary file 1 [file jcm-09-01175-s001.pdf]

Journal of
Clinical Medicine

Supplementary data

Table S1: Statistical significance and effect of cytokines from cluster #1.

Cytokines from Cluster #1	Significance	Effect	Cytokines from Cluster #1	Significance	Effect
6CKine	NS	-	IL-33	NS	-
EGF	NS	-	IL-4	NS	-
Endothelin-1	NS	-	IL-5	NS	-
Flt-3L	NS	-	IL-7	NS	-
Fractalkine	NS	-	IL-9	NS	-
G-CSF	NS	-	Leptin	NS	-
GM-CSF	NS	-	MCP-2	NS	-
I-309	NS	-	MCP-4	NS	-
IFNa2	NS	-	MDC	NS	-
IFNy	NS	-	MIP-1a	NS	-
IL-12P40	NS	-	MIP-1B	NS	-
IL-12P70	NS	-	MIP-1d	NS	-
IL-13	NS	-	OC	NS	-
IL-15	NS	-	PHT	NS	-
IL-17A	NS	-	SCF	NS	-
IL-1a	NS	-	SDF-1a+B	NS	-
IL-1B	NS	-	TARC	NS	-
IL-1RA	NS	-	TGF-a	NS	-
IL-2	NS	-	TNFa	NS	-
IL-20	NS	-	TNFB	NS	-
IL-21	NS	-	TPO	NS	-
IL-23	NS	-	TSLP	NS	-
IL-28A	NS	-	VEGF-C	NS	-
IL-3	NS	-			

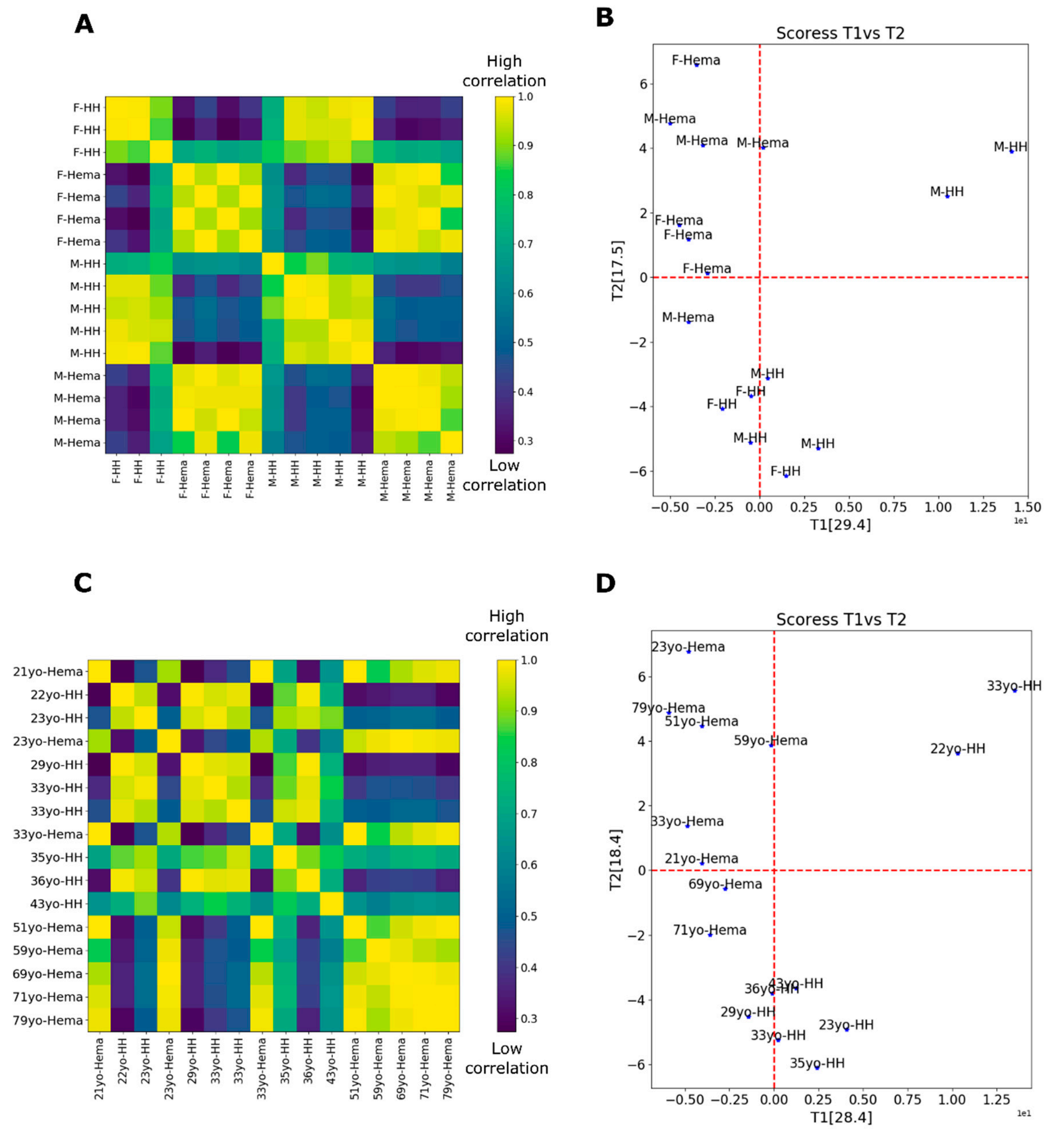

Figure S1. (**A**) Correlation matrix of ELISA multiplex data from serum of 8 healthy donors (HH-#) versus 8 hematoma extracts from pelvic fractures (Hema-#) organized in respect to their sex. (**B**) PCA analysis of ELISA multiplex cytokine dosage showing the scores depicted as individuals according to their sex. (**C**) Correlation matrix of ELISA multiplex data from 8 healthy donor serum (HH-#) versus 8 hematoma extracts from pelvic fracture (Hema-#) organized in respect to their age. (**D**) PCA analysis of ELISA multiplex cytokine dosage showing the scores depicted as individuals according to their age.

© 2020 by the authors. Licensee MDPI, Basel, Switzerland. This article is an open access article distributed under the terms and conditions of the Creative Commons Attribution (CC BY) license (http://creativecommons.org/licenses/by/4.0/).
